# Routes Obey Hierarchy in Complex Networks

**DOI:** 10.1038/s41598-017-07412-4

**Published:** 2017-08-03

**Authors:** Attila Csoma, Attila Kőrösi, Gábor Rétvári, Zalán Heszberger, József Bíró, Mariann Slíz, Andrea Avena-Koenigsberger, Alessandra Griffa, Patric Hagmann, András Gulyás

**Affiliations:** 1 Budapest University of Technology and Economics, Dept. of Telecommunications and Media Informatics, MTA-BME Information Systems Research Group, H-1117 Budapest, Magyar tudósok krt. 2, Hungary; 2 Eötvös Loránd University, Institute of Hungarian Linguistics and Finno-Ugric Studies, H-1088 Budapest, Múzeum krt. 4/A, Hungary; 3 Indiana University, Psychological and Brain Sciences, Bloomington, IN 47405 USA; 4 Department of Psychiatry, Brain Center Rudolf Magnus, University Medical Center Utrecht, Utrecht, Netherlands; 5 Department of Radiology, Centre Hospitalier Universitaire Vaudois (CHUV) and University of Lausanne (UNIL), Lausanne, Switzerland; 6 Signal Processing Laboratory 5 (LTS5), École Polytechnique Fédérale de Lausanne (EPFL), Lausanne, Switzerland

## Abstract

The last two decades of network science have discovered stunning similarities in the topological characteristics of real life networks (many biological, social, transportation and organizational networks) on a strong empirical basis. However our knowledge about the operational paths used in these networks is very limited, which prohibits the proper understanding of the principles of their functioning. Today, the most widely adopted hypothesis about the structure of the operational paths is the shortest path assumption. Here we present a striking result that the paths in various networks are significantly stretched compared to their shortest counterparts. Stretch distributions are also found to be extremely similar. This phenomenon is empirically confirmed on four networks from diverse areas of life. We also identify the high-level path selection rules nature seems to use when picking its paths.

## Introduction

The implicit “shortest path” assumption, meaning that the used communication path in a network is the one with the shortest length, seems to dominate the network science community and most of the fundamental network metrics (diameter, average path length, centrality metrics^1^, etc.) are computed using this assumption. There are other works supposing various models^2^, network metrics (e.g. degree, centrality, congestion, homophily^3–5^) and hidden structures (e.g. hidden hierarchies and metric spaces^6–8^) guiding path selection. The contribution of these works is considerable in the modeling and understanding of simple routing strategies that can recover near shortest paths without requiring global knowledge of the topology. However, a lack of confirmation with empirical data leaves an important question open: What kind of paths are *actually* chosen by nature in real world networks?

**Figure 1:**
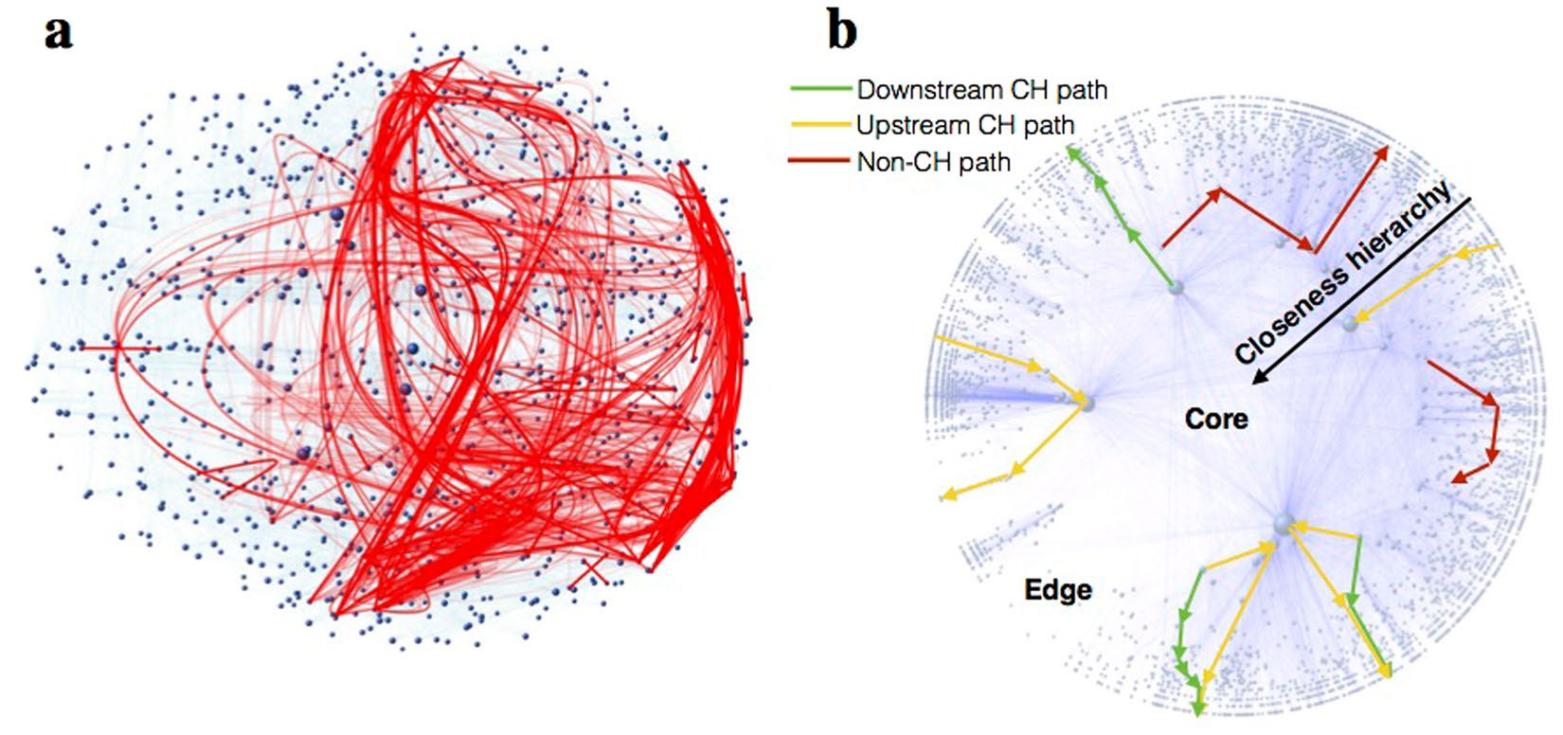
Empirical paths in the human brain (panel (a)) and the illustration of paths conforming to the policies identified by our measurements (panel (b)). A path is hierarchically conform (CH) if does not contain a large-small-large pattern forming a “valley” anywhere in its closeness centrality sequence (green and yellow paths in panel (b)). An upstream path contains at least one step towards the core of the network (yellow paths), while in downstream paths the closeness centrality monotonically decreases (green paths). The underlying faded network in panel (b) is only for illustration purposes, where smaller radial coordinate of a node indicates higher closeness centrality.

Here we approach the question of path selection in networks from this lacking empirical angle. Using existing and newly created datasets of the traffic flow on real world networks (see Methods), we compare the topology of the networks to the structure of empirically-determined paths extracted from these datasets. From this comparison we infer common characteristic rules of path selection in different networks, which we call *routing policies*. Our study here presents the analysis of empirically-determined paths in air transportation networks, the Internet, the fit-fat-cat word morph game, and empirically-inferred paths in the human brain (see Fig. 1a for an illustration). For the remainder of this text we will refer to empirically-determined and inferred paths as empirical paths. The main topological features of our networks and the statistics of the empirical paths are shown in Table 1.

**Table 1:** Basic structural properties of our networks and paths we have analyzed.

Network	Airport	Intern.	Brain	fit-fat-cat
# Nodes	3433	52194	1015	1015
# Edges	20347	117251	12596.2	8320
Avg. deg.	11.85	4.49	24.82	16.39
Avg. clust.	0.64	0.32	0.42	0.44
Avg. dist.	3.98	3.93	2.997	3.52
Diam.	13	11	6.4	9
# Emp. paths	13722	2422001	394072	2700
Path avg. dist.	4.67	4.21	4.16	3.82

**Figure 2:**
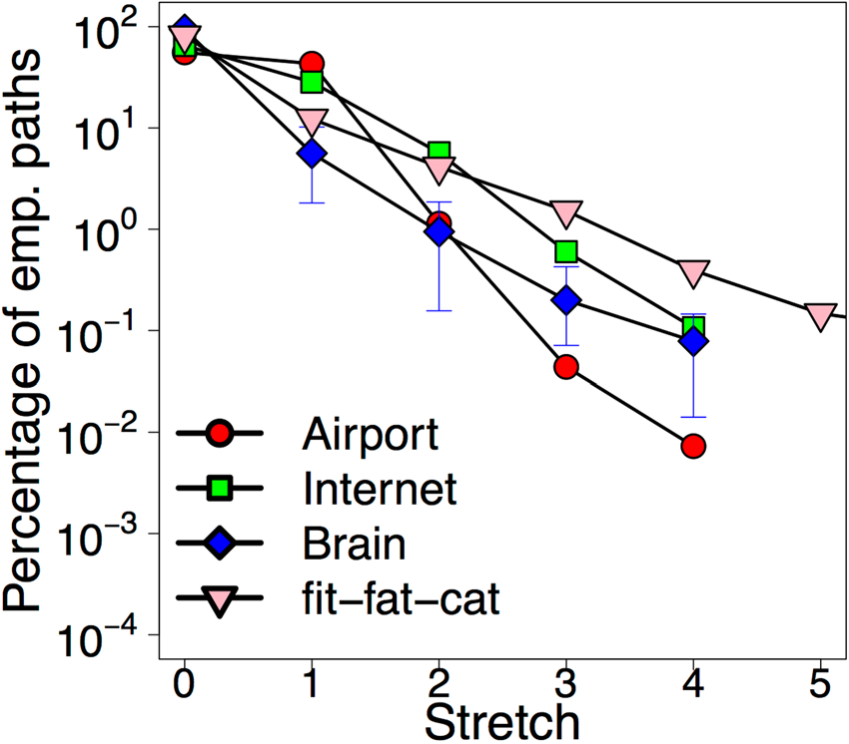
Stretch of the empirical paths with respect to their shortest counterparts. While most of the empirical paths exhibit zero stretch (confirming the shortest path assumption), a large fraction (20–40%) of the paths is “inflated” even up to 4–5 hops. The plot indicates a stunning resemblance in the distribution of path stretch in our networks.

Our first finding is that traffic in networks does not necessarily follow shortest paths. Figure 2 presents the stretch of the paths which is computed as the length of the empirical path minus the length of the corresponding shortest path having the same source and destination pair. The figure shows a significant resemblance in the distribution of path stretch across our networks. While around 60–80% of the empirical paths exhibit zero stretch, the remaining paths show path stretch which can exceed up to 4–5 hops. From this result two things follow. First, the plot confirms the shortest path assumption of previous studies in the sense that most of the empirical paths are shortest indeed. In this respect nature’s routing policy definitely “prefers short paths”. However, the non-negligible portion (20–40%) of inflated paths suggests that there may be other policies at use simultaneously.

**Figure 3:**
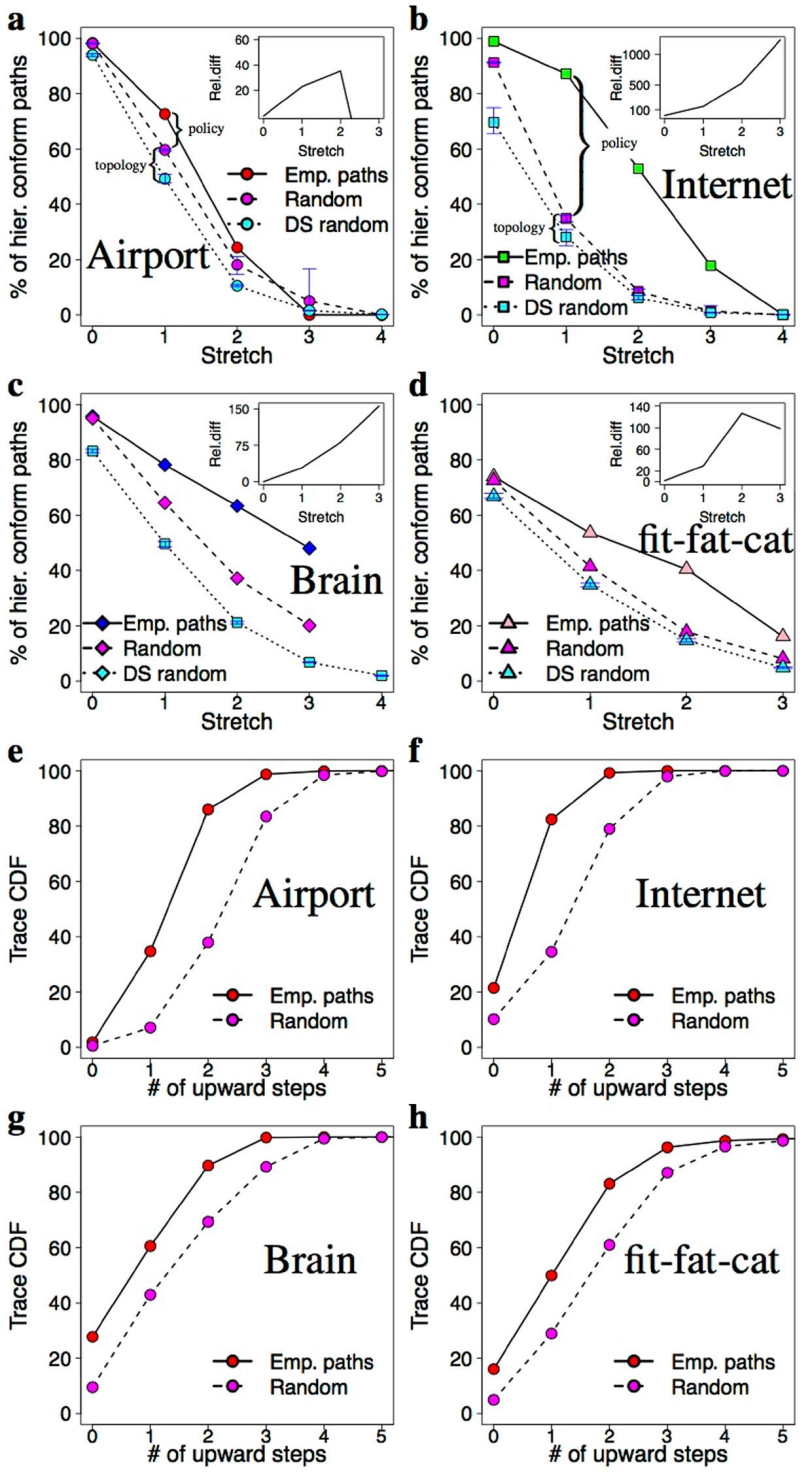
Identified routing policies confirmed by our measurement data. Panels (a–d) show the hierarchical conformity of the empirically-determined paths against stretch. The inset of the plots shows the relative difference between the number of CH paths in the empirical and the random paths. In the case of small networks there are 15–85% more CH paths in the empirical traces but in the case of the large AS level Internet this goes up to 100–500%. The cyan colored data in the plots show the number of CH paths in a randomized version of our networks generated with the degree sequence (DS) algorithm which produces exactly the same degrees for the nodes but the edges are completely randomized. The plots confirm that the topological peculiarities of real networks increase the number of CH paths between endpoints with respect to the DS networks (see the explanation brackets between the cyan and magenta colored dots of panel (a,b)). However, we argue that the effect of the CH policy is at least that important or even more fundamental (e.g. in case of the Internet). Panels (e–h) show the cumulative distribution of upstream steps in the traces of our datasets. The empirical paths tend to avoid stepping towards the core, which is reflected by the much lower number of upstream steps (in comparison with the randomly selected CH paths of the same length) before entering the downstream phase.

One such policy our measurements support is the “conform hierarchy” (CH) policy, meaning that the used paths follow the topological hierarchy of the network. For showing this we have computed the closeness centrality of the nodes comprising the empirical paths indicating which (inner or outer) parts of the network the information flows through. The closeness centrality of the node is computed as: $C(x)=\tfrac{N}{{\sum }_{y}\,d(y,x)}$, where *d*(*y*,*x*) is the distance between vertices x and y, while *N* refers to the number of nodes in the network. We found that most of the empirical paths do not contain a large-small-large pattern forming a “valley” anywhere in their closeness centrality sequence. This informally means that higher level nodes do not prefer the exchange of information through their subordinates even if there are short paths through them. On a CH path the closeness centrality increases monotonically at first up to a point (upstream), then starts to decrease (downstream) until it reaches the destination, or it is just go upstream or downstream all the way. Figure 1b illustrates this graphically. One could argue that maybe short paths on real networks have this property as a default, but Fig. 3a–d verify that this is not the case. For comparison we picked random paths between the source-destination pairs of our empirical paths with the same stretch distribution and plotted the results for that case too. One can see that, while the path length distribution is the same for the two datasets, a much larger fraction of stretch-equivalent random paths violate the CH policy.

There can be subtle differences between CH paths of similar length. For example, a path can contain upstream then downstream steps or downstream steps only. Recall that an upstream step goes towards the core, while a downstream step goes towards the periphery of the network. Is there a preference among these? For answering this we plotted the Cumulative Distribution Function (CDF) of CH paths with respect to the number of upstream steps preceding the downstream phase (Fig. 3e–h). For comparison we have also plotted the results of a random policy which picks randomly from the possible CH paths of the given length. The plots confirm that the empirical paths contain much less upstream steps, which means that these paths try to avoid stepping towards the core. This finding adds “prefer downstream” as a third identifiable policy component (see Fig. 1b for an illustration). We note that such behaviour is easy to interpret on the Internet, since stepping towards the core of the network implies paying for a transit provider for carrying the traffic, while going downstream comes for (almost) free. However, at this time it is not clear what causes the same behaviour in the other networks.

**Figure 4:**
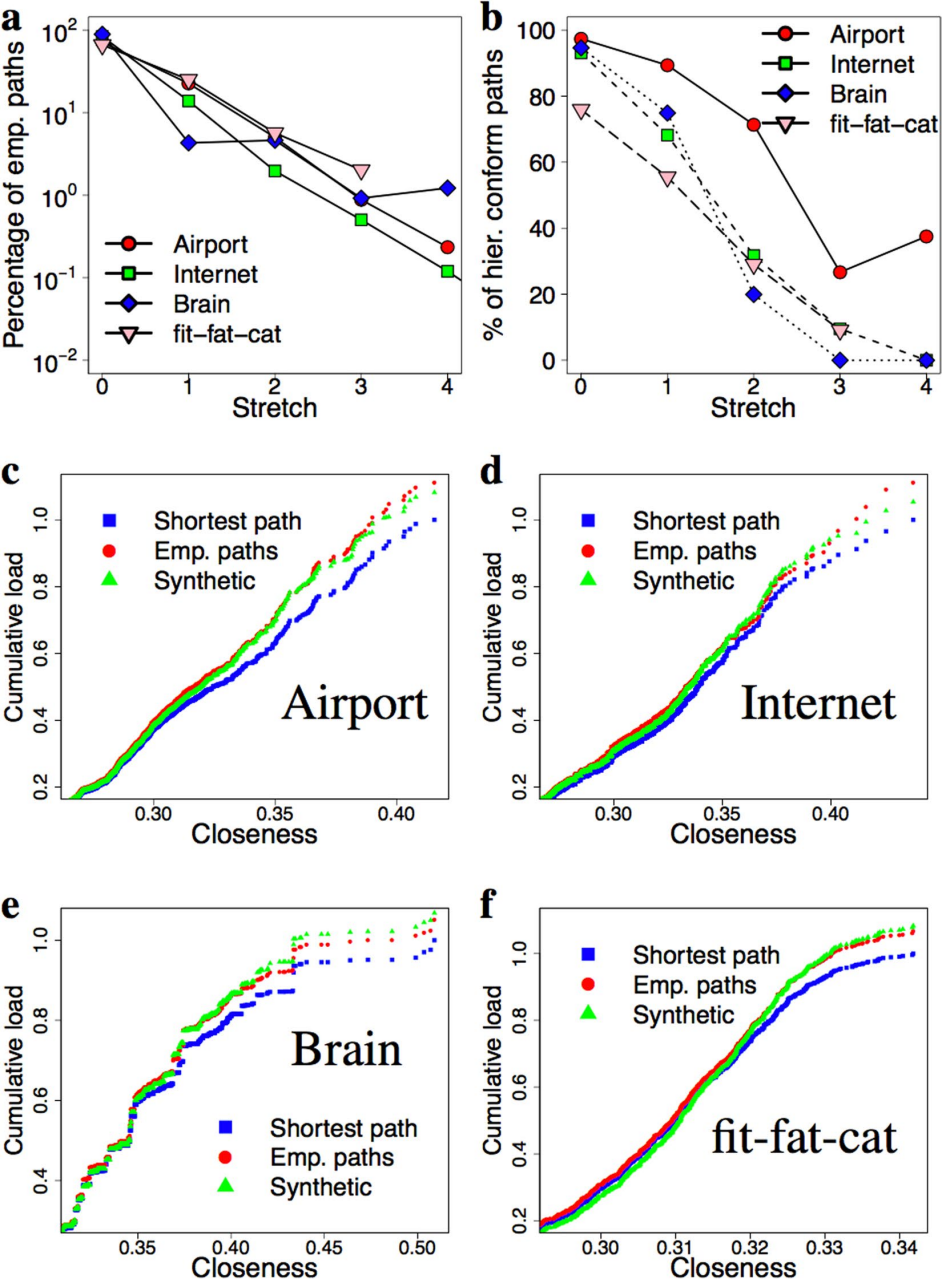
Results of the synthetic routing policy. Our toy policy exhibit very realistic stretch (panel (a)) and CH distribution (panel (b)) (see Figs 2 and 3a–d for comparison). Panels (c–f) present the cumulative load experienced on the nodes as the function of closeness, for our four datasets. The blue squares and the red circles show the load footprint of the shortest path policy and the empirical paths respectively. Due to the stretch of the empirical paths, the empirical plots give larger load that is more concentrated on the core. Our synthetic algorithm (green triangles) approximates this behaviour better than pure shortest paths.

Our datasets hint at the operation of the “prefer short paths”, “conform hierarchy” and “prefer downstream” policies, with no clear relative priorities among them. In what follows we set up a synthetic toy routing policy which uses these components in a prioritized fashion and, we show that using these simple ingredients we can approximate the empirical paths much better than simple shortest paths do. According to Fig. 2 the prefer shortest path policy can only have lower priority than the “conform hierarchy” and the “prefer downstream” otherwise we would not experience stretch at all. Since “prefer downstream” implies the conform hierarchy policy, the only reasonable choice is to: prefer hierarchically conform paths at first, then prefer downstream if there is a downstream path and from the remaining paths prefer the short paths. More specifically we define our synthetic routing policy to (*i*) use CH paths only, (*ii*) use downstream if applicable and from the paths remaining after (*i*) and (*ii*) use the shortest one (if there are still multiple choices break ties randomly). We note that such routing policy is not unfamiliar in the literature^9, 10^. Figure 4a shows that this simple routing policy immediately gives very realistic path inflation, close to the stretch computed for the real paths. What this simple algorithm cannot reproduce is that the empirical paths sometimes violate the “conform hierarchy” and the “prefer downstream” policies. Figure 4b shows the CH distribution of the paths generated with our synthetic routing policy over the same network, when the closeness values are slightly randomized. This randomization can be interpreted as simulating the case in which nodes do not have full global information about the network and therefore, the precise closeness values of the nodes are not known. Instead the nodes can only have an approximate picture about the closeness hierarchy. One can see that this modification recovers both the stretch and CH distributions exhibited by the empirical paths (see Figs 2 and 3a–d).

An immediate application of these findings is the more accurate estimation of the network’s response to outer stress (e.g. sudden load increase). Figure 4c–f show the load of the nodes when carrying the traffic of the empirical paths in comparison with shortest paths and our synthetic toy policy. One can see that real traffic has larger volume (due to the stretch) and it is even more concentrated in the core of the network. Our synthetic routing policy recovers this behavior, thus, compared to a simple shortest path policy, the synthetic policy can more accurately assess the load footprint of real paths on the network.

## Discussion

Despite the simplicity and intuitive nature of the identified policies, readers may have the feeling that there should be a more simple explanation out there. For example one could imagine a weighting of the edges over which shortest path computation will give exactly the same paths that we find in our data. We had two reasons for going this way. First, our data hints for policies that are not unfamiliar in the literature. For example, the authors of ref. 9 use a very similar routing algorithm for modeling the flow of information in organizational networks, after an in-depth analysis of such networks. Secondly, we have run experiments to find an appropriate weighting using edge betweenness (see Supplementary Note 1 for a brief listing of the results). These experiments suggest that finding reasonable weights that are able to generate paths matching all of our statistics is far from trivial.

## Methods

Collecting or inferring paths in networks is a non-trivial problem. Here we list our methods for every specific networks analyzed in our paper.

### Internet AS topology and real AS paths

The Internet protocols permit the tracing of packets. We have downloaded an AS level Internet topology and full AS level packet traces from CAIDA (Center for Applied Internet Data Analysis^11^, recorded on 09/29/2015. The topology contains 52194 nodes and 117251 connections. For this topology we had around 2.5 million traces.

### Air transportation network and flight travels

The world’s flight map is available from OpenFlights^12^, from which the topology of the air transportation network can be reconstructed. For a realistic estimation of the flights used by customers, we used the Rome2Rio^13^ trip planner and generated routes between 27444 randomly chosen pairs of airports. From the offered paths we have chosen the cheapest one in the analysis in the paper. However, we note that picking according to other parameters (lowest number of transfers, lowest travel time) did not qualitatively change our results. To achieve a more realistic topology we used airport connections extracted from traceroutes to increase the accuracy of the OpenFlight topology. The reconstructed map contained 3433 airports and 20347 flights connecting them.

### fit-fat-cat word ladder game app and word chains

For collecting paths from word networks we have implemented a word ladder game named “fit-fat-cat” for smartphones. The goal of the game is to transform a source word into a target word through meaningful intermediate words by changing only one letter at a time. The word chain fit-fat-cat is a good solution of a game with source word fit and target word cat. These word chains, collected anonymously from our users, can be considered as the footprints of human navigation over the word-maze of the English language. For the reconstruction of the word graph we have downloaded the official three-letter English Scrabble words from WordFind^14^ and created an edge between all the words differing only in one letter. The collected three-letter word chains were considered as our traces. For capturing only the “working” paths we have filtered out the first 20 games (the warming up phase) and the games taking more than 30 seconds (when the players are not just using a known path but discover an unknown one) of every player. After all, we have a dataset of more than 2500 paths from 100+ players.

### Human brain and estimated paths

Getting realistic paths from inside the human brain is extremely hard, if not impossible. As a consequence, almost all studies in the literature concerning path-related analysis assume shortest path signaling paths. Taking into account the extreme non-triviality of path estimation in the brain we ask here if we can use empirical anatomical and functional data to infer feasible communication traces. Our dataset comprises 40 healthy human subjects who underwent an MRI session where Diffusion Spectrum Imaging (DSI) and resting-state functional MRI data were acquired for each subject. DSI data was processed following the procedures described in refs 15–17, resulting in 40 weighted, undirected structural connectivity maps (*GS*) comprising 1015 nodes, where each node represents a parcel of cortical or subcortical gray matter, and connections represent white matter streamlines connecting a pair of brain regions. Connection weights determine the average density of white matter streamlines and here only consider connections with density above 0.0001, resulting in *GS* with an average of 12596.2 connections per subject. Functional MRI data was processed following state of the art pipelines described in refs 18 and 19, yielding a BOLD signal time-series per node, each with 276 points that were sampled every 1920 ms. The magnitude of the BOLD signal is an indicator of the degree of neural activity at a node. Combining structural and functional data, we infer feasible structural pathways through which neural signals might propagate using the following process. (*i*) Identify source-destination pairs with high statistically-dependent brain activity. We searched for pairs of nodes such that the Pearson correlation of the BOLD signal time series - without global regression - was above 90%. These nodes were used as the source-destination pairs of our paths. (*ii*) Determine which nodes are active at every time-step. We say that a node is “active” at a given time-step if the BOLD signal is >*γ* and “inactive” otherwise. We construct activity vectors for each time-step indicating which nodes were active. Here we use *γ* = 0 but we get qualitatively similar results for near zero *γ*. (*iii*) Construct subgraphs of active nodes. We constructed a subgraph *GS*_*i*_ of *GS* for each time-step by considering only the nodes that are active at a given time-step *i*. (*iv*) Define paths between source-destination node pairs. For all of our source-destination pairs (generated in step (*i*)), we considered the shortest path in the *GS*_*i*_ graphs, if the path existed. If there was multiple shortest paths between a source-destination pair we choose one randomly. Our source-destination traces include the paths found across all *GS*_*i*_ subgraphs. It is worth noting that this method assumes that information can only traverse active nodes. Furthermore, we are considering here a model for large spatial and temporal scale communication in brain networks that is not necessarily applicable to neural networks at smaller scales. While we cannot validate with empirical data whether these paths are actually used for the flow of neural signals, from a path inflation perspective we can consider these paths as a lower bound on the length of the real signaling pathways.

### Data Availability

The data supporting the findings of this study are either available from public data repositories or can be requested from the authors via e-mail. In particular, the topology of the AS level Internet and the corresponding AS traceroutes are available at CAIDA (Center for Applied Internet Data Analysis, www.caida.org). We have downloaded the airport network from the OpenFlights database (www.openflights.org), while the reconstruction of the word graph was based on the list of official three-letter English Scrabble words from WordFind^14^. The remaining datasets (air, brain, fit-fat-cat paths and the structural and functional brain dataset) can be requested via e-mail to the corresponding author.

## Electronic supplementary material

Supplementary Information
