# Supplementary material for: Routes Obey Hierarchy in Complex Networks
Source: Sci Rep. 2017 Aug 3;7:7243. doi: 10.1038/s41598-017-07412-4 (PMC5543142; doi:10.1038/s41598-017-07412-4)
Supplement: Supplementary file 1 — Supplementary Information [file 41598_2017_7412_MOESM1_ESM.pdf]

Supplementary information for: Routes Obey Hierarchy in Complex Networks

Attila Csoma, Attila Kőrösi, Gábor Rétvári, Zalán Heszberger,
József Bíró, Mariann Slíz, Andrea Avena-Koenigsberger, Alessandra Griffa,
Patric Hagmann, András Gulyás
Corresponding author: András Gulyás (gulyas@tmit.bme.hu)

Supplementary Figures

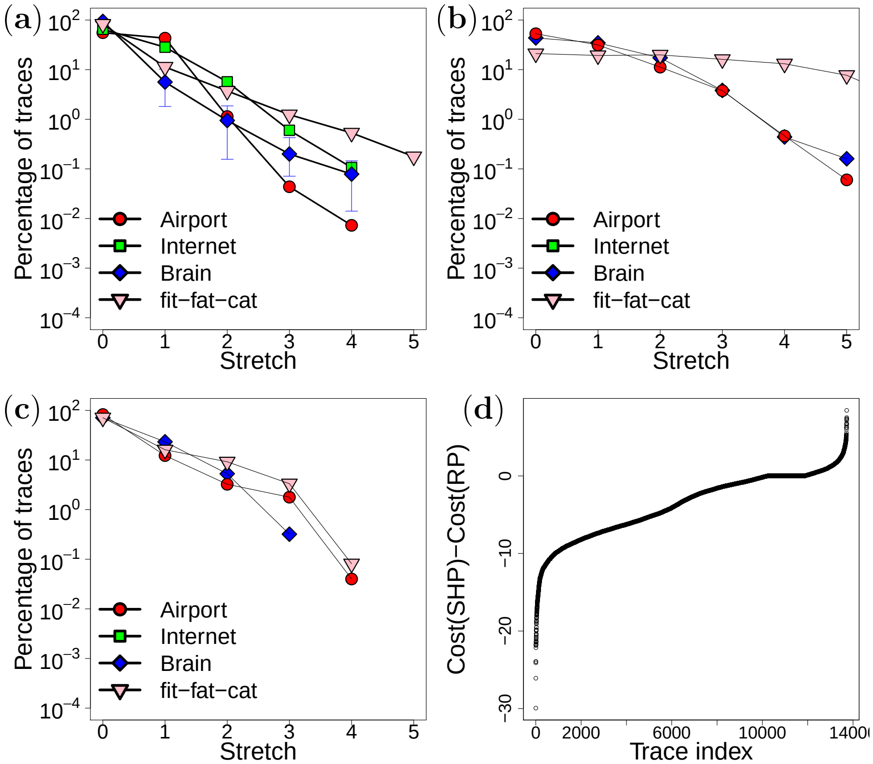

Figure 1: Results with betweenness based policies. Panel (**a**) replicates the stretch data from real networks. Panel (**b**) shows the stretch results of an experiment where all link weights are set to $\frac{1}{\mathrm{EB}_i}$ and shortest paths are generated using these weights. Stretch distribution by using $\log(\frac{\max(\mathrm{EB})}{\mathrm{EB}_i})$ as link weights is presented in panel (**c**). This plot matches better with the real data on panel (**a**). However, panel (**d**) indicates that this setting cannot be the cause of stretch in real networks, since in this weighting the sum of weights on the real paths is larger then of the simple hop-based shortest paths where all weights are set to one.

Supplementary Note - 1

The researchers' desire towards simplicity would suggest that there should be a simple weighting of the edges over which shortest path computation can recover all the observed statistics of real paths. Although we cannot disprove that such weighting exists, we list here some of our experiments to illustrate that obtaining the correct weights is far from trivial. In our first attempt we set the weights to $w_i = \frac{1}{\mathrm{EB}_i}$, where EB stands for egde betweenness. This setting reflects the intuition that edges with high betweenness are "cheaper" or "less congested" or "have more capacity" and using them is better than using hop based shortest paths (where $w_i = 1$). Panel (**b**) of Figure 1 shows that computing the shortest paths over this weighting results in the appearance of stretch indeed. However the stretch in this case is way higher than we experience

in real networks. In this setting $50-60\%$ of the paths are inflated in contrary with our real data which exhibit stretch only for $\approx 30\%$ of the real paths. Even the decay of the stretch distribution is clearly different than the real data replicated in panel (**a**) of Figure 1. We have also tried the setting $w_i = \frac{1}{\sqrt{\mathrm{EB}_i}}$ which produces a very similar plot.

We could recover very realistic stretch distribution by applying the weighting: $w_i = \log(\frac{\max(\mathrm{EB})}{\mathrm{EB}_i})$ (see panel (**c**) in Figure 1). This setting is based on the observation that the availability of edges with high betweenness is larger i.e. edges in the core are more reliable. Computing shortest paths over these weights gives a path that is available with the highest probability. Although the stretch distribution is very promising in this case panel (**d**) points that such weighting cannot be the reason for stretch in real networks. The plot shows $\sum_{i \in SHP} w_i - \sum_{i \in RP} w_i$ for all source target pairs of our real traces, where SHP stands for the hop based shortest path using $w_i = 1$ and RP stands for the real paths. In 80% of the cases the real path is longer that the hop based shortest path (where the plot is negative). This means that the hop based shortest path is actually better in this weighting that the real paths.
